# Treatment of dissociative disorders and reported changes in inpatient and outpatient cost estimates

**DOI:** 10.1080/20008198.2017.1375829

**Published:** 2017-09-19

**Authors:** Amie C. Myrick, Aliya R. Webermann, Willemien Langeland, Frank W. Putnam, Bethany L. Brand

**Affiliations:** ^a^ Family and Children’s Services, Bel Air, MD, USA; ^b^ Department of Psychology, Towson University, Towson, MD, USA; ^c^ Bascous, France; ^d^ School of Medicine, University of North Carolina, Chapel Hill, NC, USA

**Keywords:** Dissociative disorders, treatment costs, hospitalization, stages of treatment

## Abstract

**Background:** Interpersonal trauma and trauma-related disorders cost society billions of dollars each year. Because of chronic and severe trauma histories, dissociative disorder (DD) patients spend many years in the mental health system, yet there is limited knowledge about the economic burden associated with DDs.

**Objective:** The current study sought to determine how receiving specialized treatment would relate to estimated costs of inpatient and outpatient mental health services.

**Method:** Patients’ and individual therapists’ reports of inpatient hospitalization days and outpatient treatment sessions were converted into US dollars. DD patients and their clinicians reported on use of inpatient and outpatient services four times over 30 months as part of a larger, naturalistic, international DD treatment study. The baseline sample included 292 clinicians and 280 patients; at the 30-month follow-up, 135 clinicians and 111 patients. Missing data were replaced in analyses to maintain adequate statistical power. The substantial attrition rate (>50%) should be considered in interpreting findings.

**Results:** Longitudinal and cross-sectional analyses of cost estimates based on patient reported inpatient hospitalization significantly decreased over time. Longitudinal cost estimates based on clinician-reported outpatient services also significantly decreased over time. Cross-sectional cost estimates based on patient and clinician reported inpatient hospitalization were significantly lower for patients in later stages of treatment compared to those struggling with safety and stabilization. Cross-sectional cost estimates based on clinician-reported outpatient services were significantly lower for patients in later stages of treatment compared to those in early stages.

**Conclusions:** This pattern of longitudinal and cross-sectional reductions in inpatient and outpatient costs, as reported by both patients and therapists, suggests that DD treatment may be associated with reduced inpatient and outpatient costs over time. Although these preliminary results show decreased mental health care utilization and associated estimated costs, it is not clear whether it was treatment that caused these important changes.

## Abstract (es)

**Planteamiento:** El trauma interpersonal y los trastornos relacionados con el trauma cuestan a la sociedad miles de millones de dólares cada año. Debido a las historias de trauma crónico y grave, los pacientes con trastorno disociativo (TD) pasan muchos años en el sistema de salud mental, sin embargo, hay escasez de conocimiento sobre la carga económica asociada con los TDs.

**Objetivo:** El presente estudio trató de determinar cómo recibir tratamiento especializado se relacionaría con los costos estimados de los servicios de salud mental para pacientes hospitalizados y ambulatorios.

**Método:** Los informes de los pacientes y los terapeutas de los días de hospitalización y las sesiones de tratamiento ambulatorio se convirtieron a dólares estadounidenses para determinar los cambios en el costo estimado. Los pacientes con TD y los clínicos informaron sobre el uso de servicios de hospitalización y ambulatorios cuatro veces durante 30 meses como parte de un estudio de tratamiento de TD más extenso, naturalista e internacional. La muestra de referencia incluía 292 clínicos y 280 pacientes; el seguimiento a los 30 meses incluyó a 135 clínicos y 111 pacientes. Los datos faltantes se reemplazaron en los análisis para mantener una fuerza estadística adecuada. La tasa de desgaste sustancial (>50%) debe tenerse en cuenta en la interpretación de los resultados.

**Resultados:** Los análisis longitudinales y transversales de las estimaciones de costos –basados en las hospitalizaciones referidas por los pacientes, disminuyeron significativamente con el tiempo. Las estimaciones de costos longitudinales –basadas en servicios ambulatorios referidos por el clínico– también disminuyeron significativamente con el tiempo. Las estimaciones transversales de costos basadas en informes de hospitalización del paciente y el clínico fueron significativamente más bajas para los pacientes en etapas posteriores de tratamiento en comparación con aquellos que estaban lidiando con temas de seguridad y estabilización. Las estimaciones de costos transversales basadas en los servicios ambulatorios referidos por el clínico fueron significativamente menores para los pacientes en etapas posteriores del tratamiento en comparación con las etapas más tempranas del tratamiento.

**Conclusiones:** Este patrón de reducciones longitudinales y transversales en los costos de los pacientes hospitalizados y ambulatorios sugiere que el tratamiento de los TD puede estar asociado con costos reducidos de pacientes hospitalizados y ambulatorios a lo largo del tiempo. Aunque estos resultados preliminares muestran una disminución en el uso de servicios de salud mental y los costos estimados asociados, no está claro si fue el tratamiento el que causó estos cambios importantes

## Abstract (zh)

**背景：**人际创伤和创伤相关的障碍每年花掉社会数以亿计的美元。因为创伤历史的长期性和严重性，分离障碍（DD）病人需要在精神健康系统里治疗许多年，但仍然缺少对DD经济负担的了解。

**目标：**本研究想要了解接受专业治疗和住院、门诊病人的精神治疗服务费用的关系。

**方法：**病人和治疗师报告住院病人住院天数和门诊病人的治疗疗程，并将其转化为以美元为单位来估算花费的变化。在30个月里，DD病人和临床医生报告四次其使用住院、门诊服务的情况（作为一个更大型的、国际化的DD治疗自然研究的一部分）。基线样本包括292名临床医生和280名病人；30月追踪阶段包括135名临床医生和111名病人。在分析中插补缺失数据，以保证足够的统计效力。在解释结果时不能忽视较大的流失率（>50%）。

**结果：**基于住院病人报告的住院情况，对费用估算的追踪和横向分析发现其随着时间显著减少。根据临床医生报告的门诊病人的服务，长期费用估算也随着时间显著减少。根据病人和临床医生报告的住院病人的入院进行横向费用估算，治疗后期的病人显著少于还在安全期和稳定期的病人。基于临床医生报告的门诊病人服务进行横向花费估计，治疗的后期病人也显著低于治疗早期病人。

**结论：**这种在住院、门诊病人中长期和横向费用减少的模式提示DD治疗可能和住院、门诊病人的费用减少有关。尽管这些初步结果显示精神健康服务使用和相关费用的消减，依然不清楚是否是由治疗导致了这些关键变化。

## Highlights

Trauma and dissociative disorders cost society billions of dollars each year. Dissociative disorder clients typically spend many years in treatment. Many are hospitalized repeatedly over time.

Most of the longitudinal cost estimates based on patient- and therapist-reported inpatient hospitalizations significantly decreased over 30-months of trauma- and dissociation-focused treatment, as did most of the longitudinal cost estimates based on clinician-reported outpatient services.

The overall pattern of cross-sectional cost estimates based on patient- and clinician-reported inpatient hospitalization also showed significantly lower costs for patients in later stages of treatment compared to those in early stages of treatment. At 18 months, cross-sectional cost estimates based on therapist-reported outpatient sessions showed significantly lower costs for patients in later stages of treatment compared to those in early stages of treatment.

Research has increasingly considered the cost of mental health issues among the general population but, to date, very few studies have considered the economic impact of trauma-related disorders. The limited research conducted has revealed that interpersonal trauma substantially costs society. In their review of economic costs of domestic violence, Waters and colleagues  (2004) found that domestic violence costs the U.S. as much as 12.6 billion USD annually. Other studies have estimated that child abuse and neglect costs the U.S. over 100 billion USD each year (Wang & Holton, 2007) and, internationally, the cost of childhood maltreatment ranges between 11.1 billion and 29.8 billion Euro (EUR) annually (see Habetha, Bleich, Weidenhammer, & Fegert, 2012), or the equivalent of 12.4–33.3 billion USD.^1^ The cost estimates vary widely according to the types of costs included by researchers.

A study by Ferry and colleagues (2015) estimated the annual direct costs (i.e. service visits) of posttraumatic stress disorder (PTSD) in Northern Ireland at nearly 47 million USD. However, when indirect costs such as lost productivity and days missed from work were included, this number jumped to an estimated 246 million USD (Ferry et al., 2015). In this study, hospital stays accounted for the highest annual service costs, at over 12 million USD. It is likely these costs accrue over time. Kessler (2000) suggested many individuals with PTSD experience symptoms for more than two decades, during which time associated costs to the individual and society are staggering.

Emerging research on the economic costs of trauma is particularly salient for patients with dissociative disorders (DDs). These patients’ severe and chronic trauma histories are well documented, as are the many years they spend in the mental health system receiving inpatient and outpatient services (Boon & Draijer, 1993; Fraser & Raine, 1992; Hornstein & Putnam, 1992; Lloyd, 2011; Putnam, Guroff, Silberman, Barban, & Post, 1986; Ross & Dua, 1993; Ross, Joshi, & Currie, 1990). Mansfield and colleagues (2010) reported that DD patients who were spouses of active-duty U.S. military personnel utilized mental health services at a higher rate than individuals diagnosed with any of the other 16 psychiatric disorders they studied. Furthermore, studies have found that DD patients spend an average of six to eight years in treatment before being correctly diagnosed (e.g. Middleton, 2004; Putnam et al., 1986; Rivera, 1991). During that time, they typically receive costly evaluations, partake in lengthy and ineffective treatments, and are hospitalized multiple times (e.g. Boon & Draijer, 1993; Loewenstein, 1990; Rivera, 1991). DD patients also frequently attempt suicide. Each individual suicide attempt among general psychiatric patients costs an estimated 2000–68,000 USD when direct costs such as ambulatory care, medical tests, surgeries, and psychiatric treatment are considered (reviewed in Yang & Lester, 2007). Some preliminary studies have calculated that swift and accurate diagnosis of DDs, followed by appropriate trauma- and dissociation-focused treatment, would substantially decrease the cost of DD patients’ treatments, even in cases where patients are severely impaired (Fraser & Raine, 1992; Lloyd, 2011; Ross & Dua, 1993).

Focusing on the costs incurred by those with DDs and the changes in costs over time can help researchers understand the benefits and costs ratio of interventions available to this population (Haddix, Teutsch, & Corso, 2003; Teutsch, 1992). Some authors have suggested that DD treatment is harmful (Lambert & Lilienfeld, 2007; Lilienfeld, 2007); if this viewpoint is accurate, specialized treatment would likely also increase the economic burden of DD patients on the health care system. This is an important area that merits more research.

The present study examines the changes in costs estimates associated with reports of inpatient hospitalization and outpatient sessions of patients involved in outpatient trauma- and dissociation-focused treatment over 30 months. The study utilized data gathered in the Treatment of Patients with Dissociative Disorders (TOP DD) study, which was a prospective, longitudinal, and naturalistic study of DD patients with additional data reported by their clinicians (Brand et al., 2009). Because these participants demonstrated improvements in dissociative, posttraumatic, and depressive symptoms, decreased rates of hospitalization and suicidality, and increased adaptive functioning over the course of the study (e.g. Brand & Loewenstein, 2014; Brand et al., 2013; Brand & Stadnik, 2013; Myrick et al., 2012), we hypothesized that inpatient and outpatient treatment estimated costs would decrease over time. Furthermore, we expected that patients in the earlier stages of treatment, who struggle with safety issues such as self-injurious behaviour and suicidality (e.g. Coons & Milstein, 1990; Foote, Smolin, Neft, & Lipschitz, 2008), would incur greater treatment costs compared to patients in the later stages of treatment.

Prior analyses have not examined cost estimates, although this is a topic that has important implications and is understudied. To address the need for research into DD treatment costs, we grouped patients into early treatment (stage 1–2) and late treatment (3–5) groups because early stage patients evidence greater struggles with safety, stability, and self-harm than do patients at later stages in their treatment (Brand et al., 2009; ISSTD, 2011). Thus, patients in earlier stages of treatment are likely to accrue greater inpatient and outpatient costs than patients who have stabilized and advanced to later stages of treatment.

## 1. Methods

### 1.1. Participants

Patients were diagnosed with dissociative identity disorder (DID) or dissociative disorder not otherwise specified/other specified dissociative disorder (DDNOS/OSDD). Patients and clinicians were required to have been engaged in treatment together for a minimum of three months prior to study enrolment. Clinicians provided trauma- and dissociation-focused outpatient treatment. Additional details on the study’s recruitment, methodology, and outcomes are available (Brand et al., 2009, 2013). At baseline, the sample included 292 clinicians and 280 patients; at the 30-month follow-up, the sample included 135 clinicians and 111 patients. The rate of attrition in this study was approximately 50% by 30-month follow-up, and varied by time period and patient characteristics; it was higher during the first six months and among patients with higher dissociation scores at baseline as well as those who had a substance use/dependence disorder (Brand et al., 2013). Only those who completed the last survey were considered retained. The sample was recruited internationally: 8% of participants were from Canada; 18% of participants were from 17 countries outside North America, most notably the UK and the Netherlands. However, because three-quarters (74%) of the sample was from the U.S. (*N *= 220 therapists), and due to the necessity of a constant service cost for cost analyses, U.S. standard cost values were used in the present study.

### 1.2. Data sources

Data on inpatient hospitalization and outpatient sessions from therapists were obtained from a questionnaire adapted from Zittel Conklin and Westen (2005) administered at three time points within the 30-month duration of the TOP DD study, including follow-ups at month six (T2), month 18 (T3), and month 30 (T4). Data from patients on their inpatient hospitalization days was obtained from a questionnaire adapted from the National Health and Nutrition Examination Survey (National Center for Health Statistics, n.d.) administered at four time points within the 30-month study, including at baseline (T1) as well as the three follow-ups (T2–T4). Both patients and therapists were asked about patients’ number of inpatient days, but only therapists were asked about patients’ number of outpatient sessions.

#### 1.2.1. Inpatient days

Patients were asked at four time points (T1–T4) if they had been hospitalized in a psychiatric hospital in the past six months. If they answered yes, they reported the number of days they had been hospitalized. Clinicians were asked at three follow-up time points (T2–T4) to estimate the total number of days their patient had been hospitalized in a psychiatric hospital over the past six months. Clinician reports on patients’ utilization of inpatient services were not collected at baseline (T1).

#### 1.2.2. Outpatient sessions

At each follow-up (T2–T4), clinicians were asked how many times they provided individual psychotherapy sessions for the patient in a typical month over the last six months. The number of outpatient sessions was multiplied by six to reflect the total number of outpatient sessions over a six-month time period. Clinician reports of patients’ utilization of outpatient sessions were not collected at baseline (T1).

#### 1.2.3. Cost estimates

The average costs of inpatient and outpatient services were estimated by defining what the services entailed and then assigning a corresponding USD value. Inpatient services were defined as a day of inpatient psychiatric hospitalization, priced at 713 USD/day using Medicaid and Medicare 2008 fee schedules (MedPac, 2010). Inpatient costs were computed by multiplying inpatient days in six months by 713 USD. One outpatient 40–50 minute psychotherapy session was priced at 85 USD/session by using the aforementioned Medicare and Medicaid fee schedules (Centers for Medicare and Medicaid Services, 2007). Outpatient psychotherapy costs were estimated by multiplying the number of outpatient sessions in six months by 85 USD. Medicare and Medicaid service fee schedules were used because Medicare is the standard by which many U.S. insurance companies set their reimbursement schedule for health care services.

### 1.3. Analyses

First, to assess longitudinal changes, repeated measures ANOVAs were used to assess whether there were significant mean differences in patients’ hospitalization days (via both clinician and patient report) and outpatient sessions (via clinician report) over the 30-month duration of the TOP DD study. For significant omnibus tests, comparisons were made for each time point temporally following one another (i.e. T1–T2, T1–T3, T2–T3, T2–T4, T3–T4 for patient reports; T2–T3, T3–T4 for therapist reports), as well as time points across the duration of the study (i.e. T1–T4 for patient reports, T2–T4 for therapist reports).

Second, to assess cross-sectional differences, a one-way MANOVA assessed mean differences in cost variables (inpatient and outpatient costs) among patients based on their stage of treatment, as classified by their clinician. In line with expert guidelines on the treatment of complex DDS, treatment stages in the present study included stage 1 (i.e. stabilization and establishing safety), stage 3 (i.e. processing memories of trauma with full emotion and grieving related losses), and stage 5 (i.e. integration and reconnection within self and with others; International Society for the Study of Trauma and Dissociation [ISSTD], 2011), as well as two intermediate stages (stages 2 and 4). Early-stage patients (e.g. stages 1 and 2) were combined, as were late-stage patients (e.g. stages 3–5). Combining patients by treatment stage allowed for greater sample size and subsequent statistical power of analyses. A Bonferroni correction was applied to the multiple pairwise comparisons within the repeated measures ANOVA and MANOVA analyses to adjust for alpha inflations due to multiple hypothesis testing.

#### 1.3.1. Missing data

By default, repeated measures ANOVA and MANOVA delete cases listwise in analyses, and given the attrition over the 30 month-duration of the study, notably reduced sample size and power for the analyses. Sample sizes for analyses before MI ranged from *N *= 46–93, and G*Power a priori power analyses estimated that with a small effect size (.20), an *N *= 80–152 was needed for the repeated measures ANOVA, and *N *= 386 for the MANOVA. Thus, missing data were replaced through multiple imputation, a frequently used process which replaces missing data through imputing, analysing, and pooling missing data (Schafer, 1999). Multiple imputation is a recommended process for handling missing data regardless of the type of missing data (that is, missing at random, missing completely at random, or missing not at random; Schafer, 1999). All analyses and multiple imputation procedures were conducted through IBM SPSS Statistics version 24. The sample size and descriptive statistics for variables before and after MI are given in Tables 1 and 2.

#### 1.3.2. Outliers

The data were not normally distributed and contained outliers, including but not limited to the one or two patients at each time point who were hospitalized for the entire six-month period assessed, accruing approximately 130,000 USD in inpatient costs over a six-month duration. To avoid outliers skewing the analyses, the top 5% of each cost variable was removed from cost analyses. Trimming the top 5% of outliers is a common technique used in datasets with extreme outliers (Field, 2013). In order to trim the top 5% of each cost variable, the 95th percentile was identified for each cost variable, and each variable was trimmed above this 95th percentile value, resulting in the removal of 43 outliers across all analyses (Tables 1 and 2). Some participants represented outliers within multiple cost variables, while others were an outlier within only one cost variable, and thus removal of outlier values was done on a case-by-case basis for each variable.

**Table 1.** Descriptive statistics for patient variables.

Variable	Time point	95^th^ percentile	Outliers removed	Total *N* before MI	Total *N* after MI	*M* before MI (*SD*)	*M* after MI (*SD*)	Median before MI	Median after MI	Skew before MI	Skew after MI	Range before MI	Range after MI
# days hospitalized inpatient	Time 1	$22,103 (31 days)	$22,103–$128,340 (*N* = 11)	213	274	$1078 ($3922)	$1100 ($3863)	$.00	$.00	4.13	3.48	$.00–$21,390	$.00–$21,390
# days hospitalized inpatient	Time 2	$28,628 (40 days)	$28,628,103–$130,479 (*N* = 5)	145	274	$1298 ($4378)	$1833 ($5682)	$.00	$.00	3.74	2.13	$.00–$21,390	$.00–$28,628
# days hospitalized inpatient	Time 3	$10,695 (15 days)	$10,695–$46,345 (*N* = 6)	129	274	$415 ($1741)	$426 ($1921)	$.00	$.00	4.41	1.56	$.00–$9982	$.00–$9982
# days hospitalized inpatient	Time 4	$16,399 (23 days)	$21,390–$128,340 (*N* = 6)	122	274	$251 ($1341)	$468 ($1841)	$.00	$.00	5.86	1.91	$.00–$9269	$.00–$9269

Patient reports of outpatient therapy sessions were not collected.

**Table 2.** Descriptive statistics for clinician variables.

Variable	Time point	95th percentile	Outliers removed	Total *N* before MI	Total *N* after MI	*M* (*SD*) after MI	*M* (*SD*) before MI	Median before MI	Median after MI	Skew before MI	Skew after MI	Range before MI	Range after MI
# days hospitalized inpatient	Time 1	N/A	N/A	N/A	N/A	N/A	N/A	N/A	N/A	N/A	N/A	N/A	N/A
# days hospitalized inpatient	Time 2	$22,103 (31 days)	$22,816–$130,479 (*N* = 8)	176	274	$1329 ($4522)	$1358 ($4466)	$.00	$.00	3.62	2.64	$.00–$21,390	$.00–$21,390
# days hospitalized inpatient	Time 3	$22,103 (31 days)	$22,103–$42,780 (*N* = 5)	165	274	$886 ($3815)	$1344 ($4117)	$.00	$.00	4.60	2.56	$.00–$21,390	$.00–$21,390
# days hospitalized inpatient	Time 4	$20,677 (29 days)	$21,390–$106,950 (*N* = 7)	136	274	$566 ($2185)	$879 ($2400)	$.00	$.00	4.60	2.36	$.00–$15,686	$.00–$15,686
# therapy sessions outpatient	Time 1	N/A	N/A	N/A	N/A	N/A	N/A	N/A	N/A	N/A	N/A	N/A	N/A
# therapy sessions outpatient	Time 2	$7,055 (83 sessions)	$7,140–$30,600 (*N* = 9)	174	274	$2963 ($1518)	$2864 ($1451)	$2040	$2431	.51	.40	$.00–$6630	$.00–$6630
# therapy sessions outpatient	Time 3	$6,375 (75 sessions)	$6,630–$36,720 (*N* = 8)	162	274	$2745 ($1408)	$2656 ($1580)	$2040	$2311	.54	.10	$.00–$6120	$.00–$6234
# therapy sessions outpatient	Time 4	$7,395 (87 sessions)	$7,650–$35,700 (*N* = 7)	136	274	$2625 ($1422)	$2572 ($1564)	$2040	$2128	.55	.20	$.00–$6120	$.00–$6562

Clinician reports of inpatient hospitalization days and outpatient therapy sessions were not collected at time 1.

## 2. Results

### 2.1. Descriptive statistics

Table 1 contains the descriptive statistics for patient reports of days spent inpatient and their conversions into cost variables, while Table 2 contains the descriptive statistics for clinician reports of patients’ days spent inpatient and outpatient sessions attended and their conversions into cost variables. Descriptive data of the cost variables includes means and medians, standard deviations, skew values, ranges, and outliers for each cost value over the duration of the study. Results and sample size are provided firstly for the dataset with missing data replaced through MI, and secondly for the original dataset without MI.

### 2.2. Inpatient costs estimates based on patient reports

#### 2.2.1. Cost estimates over time

**Figure 1.**
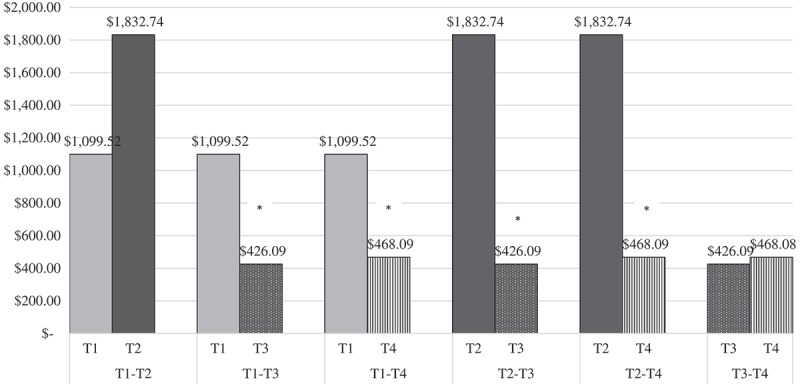
Estimated costs based on patient-reported inpatient days over time. *Significant reduction in estimated costs (*p* < .05).T1 = Time 1; T2 = Time 2; T3 = Time 3; T4 = Time 4

There was a significant mean difference in patient-reported inpatient cost estimates over time, *F*(1, 954) = 11.51, *p *< .001, partial *η^2^* = .04, *N *= 274. There were significantly lower patient-reported inpatient costs between T1 (*M* = 1099.52) and T3 (*M *= 426.09), *p *= .036, T1 and T4 (*M *= 468.08), *p *= .006, T2 (*M *= 1832.74) and T3, *p *< .001, and T2 and T4, *p *< .001 (see Table 3 and Figure 1). In the original dataset without replacement of missing data through MI, there was not a significant mean difference in patient-reported inpatient cost estimates over time, *F*(3,65) = .56, *p *= .65, *N *= 68.

**Table 3.** Repeated measures ANOVA of longitudinal inpatient and outpatient estimated cost comparisons.

Patient reports Therapist reports							
Variable	Time points (A → B)	*M* difference (A-B)	*N*	Standard error	*M* difference (A-B)	*N*	Standard error
# days hospitalized inpatient	Time 1 → Time 2	-$733.216	274	341.87	N/A	N/A	N/A
# days hospitalized inpatient	Time 1 → Time 3	$673.43*	274	243.15	N/A	N/A	N/A
# days hospitalized inpatient	Time 1 → Time 4	$631.44**	274	189.28	N/A	N/A	N/A
# days hospitalized inpatient	Time 2 → Time 3	$1406.65***	274	337.05	$14.12	274	303.35
# days hospitalized inpatient	Time 2 → Time 4	$1364.66***	274	328.35	$479.13	274	251.57
# days hospitalized inpatient	Time 3 → Time 4	-$41.99	274	1.00	$465.01	274	191.20
# therapy sessions outpatient	Time 2 → Time 3	N/A	N/A	N/A	$208.28***	274	55.78
# therapy sessions outpatient	Time 2 → Time 4	N/A	N/A	N/A	$292.87***	274	69.86
# therapy sessions outpatient	Time 3 → Time 4	N/A	N/A	N/A	$84.59	274	65.65

N/A = data not collected.

*Significant reduction in estimated costs at *p* < .05; ** *p *< .01; *** *p *< .001. Bonferroni adjustments made for all multiple comparisons.

#### 2.2.2. Cost estimates by treatment stage

**Figure 2.**
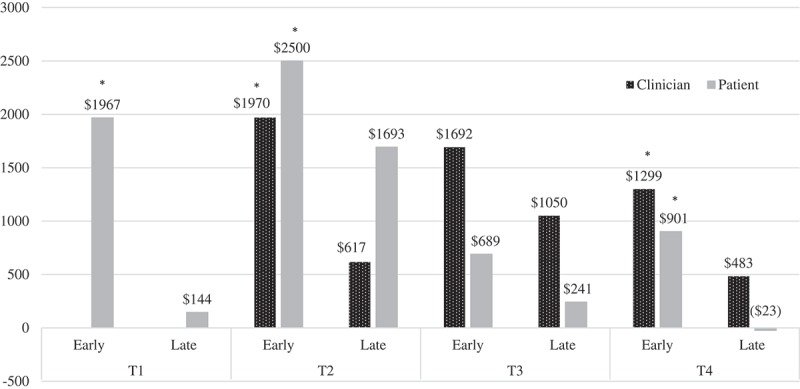
Estimated costs based on clinician- and patient-reported inpatient days by stage of treatment. *Significant estimated cost differences by stage in treatment (*p* < .05)Clinician-reported inpatient days were not collected at T1.‘Early’ = Stages 1 and 2 combined; ‘Late’ = Stages 3, 4, and 5 combinedT1 = Time 1; T2 = Time 2; T3 = Time 3; T4 = Time 4

Cost estimates differed by treatment stage for patient-reported inpatient costs, *F*(10, 234) = 2.39, *p *= .01. At T1, early-stage patients had significantly higher patient-reported inpatient costs at T1 (*M* = 1966.77) compared to later-stage patients (*M* = 144.34), *F*(1) = 13.94, *p *< .001 (see Table 4 and Figure 2). Additionally, cost estimates differed by treatment stage for T2 patient-reported inpatient costs, *F*(1) = 5.355, *p *= .02; early-stage patients had significantly higher patient-reported inpatient costs at T2 (*M* = 2499.63) compared to later-stage patients (*M* = 814.34), *p *= .02. Lastly, cost estimates differed by treatment stage for T4 patient-reported inpatient costs, *F*(1) = 14.86, *p *< .001; early-stage patients had significantly higher patient-reported inpatient costs at T4 (*M* = 900.87) compared to later-stage patients (*M* = −23.02), *p *< .001. In the original dataset without replacement of missing data through MI, there were no significant differences in patient- or clinician-reported treatment costs over T1–T4, *F*(6, 39) = .74, *p *= .55, *N *= 46.

**Table 4.** MANOVA cross-sectional inpatient and outpatient estimated cost comparisons by treatment stage.

Patient reports Therapist reports							
Variable	Time point	*F*	*N*	*M* range stages 1–2 – stages 3–5	*F*	*N*	*M* range stages 1–2 – stages 3–5
# days hospitalized inpatient	Time 1	13.94***	274	$1966.77–$144.34	N/A	N/A	N/A
# days hospitalized inpatient	Time 2	5.36*	274	$2499.63–$814.34	5.48*	274	$1969.66–$617.09
# days hospitalized inpatient	Time 3	3.19	274	$688.99–$241.09	1.42	274	$1692.67–$1049.85
# days hospitalized inpatient	Time 4	14.86***	274	$900.87–$0.00	6.66*	274	$1299.15–$483.24
# therapy sessions outpatient	Time 1	N/A	N/A	N/A	N/A	N/A	N/A
# therapy sessions outpatient	Time 2	N/A	N/A	N/A	2.03	274	$2956.11–$2687.80
# therapy sessions outpatient	Time 3	N/A	N/A	N/A	4.80*	274	$2811.85–$2370.98
# therapy sessions outpatient	Time 4	N/A	N/A	N/A	2.22	274	$2652.94–$2353.35

N/A = data not collected.

*Significant reduction in estimated costs at *p* < .05; ** *p *< .01; *** *p *< .001

### 2.3. Inpatient costs estimates based on clinician reports

#### 2.3.1. Cost estimates over time

There was not a significant mean difference in clinician-reported inpatient costs over time, *F*(1, 607) = 2.33, *p *= .110, partial *η^2^* = .008, *N *= 274. In the original dataset without replacement of missing data through MI, there was also not a significant mean difference in clinician-reported inpatient costs over time, *F*(2, 93) = 2.02, *p *= .14, *N *= 95.

#### 2.3.2. Cost estimates by treatment stage

Cost estimates differed by treatment stage for clinician-reported inpatient costs, *F*(10, 234) = 2.39, *p *= .01. At T2, early-stage patients had significantly higher patient-reported inpatient costs at T2 (*M *= 1969.66) compared to later-stage patients (*M* = 617.09), *F*(1) = 5.49, *p *= .02 (see Table 4 and Figure 2). Additionally, cost estimates differed by treatment stage for T4 clinician-reported inpatient costs, *F*(1) = 6.66, *p *= .01; early-stage patients had significantly higher patient-reported inpatient costs at T4 (*M *= 1299.15) compared to later-stage patients (*M* = 483.24), *p *= .01.

### 2.4. Outpatient costs estimates based on clinician reports

#### 2.4.1. Cost estimates over time

**Figure 3.**
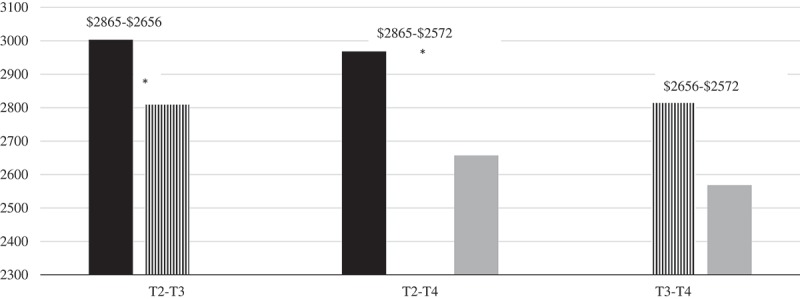
Estimated costs based on clinician-reported outpatient sessions over time. *Significant cost reduction (*p* < .05).Clinician-reported outpatient sessions were not collected at T1.Patient-reported outpatient costs not collected throughout study.T1 = Time 1; T2 = Time 2; T3 = Time 3; T4 = Time 4

There was a significant mean difference in clinician-reported outpatient cost estimates over time, *F*(1, 891) = 11.081, *p *< .001, partial *η^2^* = .04. There were significantly lower clinician-reported outpatient costs between T2 (*M *= 2864.67) and T3 (*M *= 2656.39), *p *< .001, and T2 and T4 (*M* = 2571.80), *p *< .001 (see Table 3 and Figure 3), *N *= 274. In the original dataset without replacement of missing data through MI, there was also a significant mean difference in clinician-reported outpatient cost estimates over time, *F*(2, 91) = 6.56, *p *< .02, *N *= 93.

#### 2.4.2. Cost estimates by treatment stage

**Figure 4.**
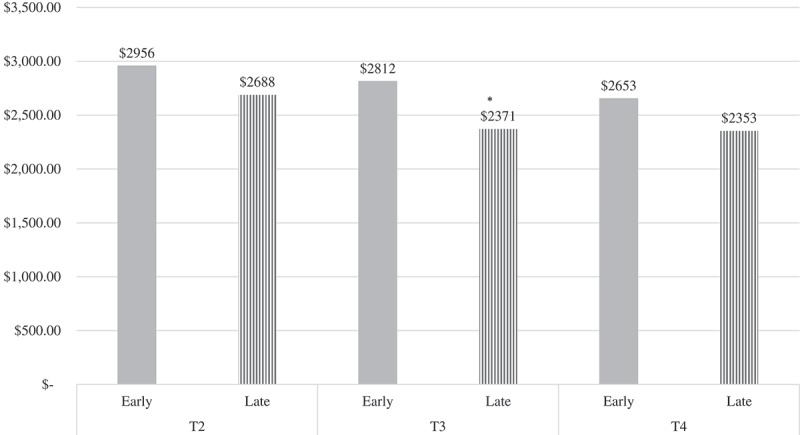
Estimated costs based on clinician-reported outpatient sessions by stage of treatment. ‘Early’ = Stages 1 and 2 combined; ‘Late’ = Stages 3, 4, and 5 combinedNo significant stage differences in outpatient costs at *p* < .05T1 = Time 1; T2 = Time 2; T3 = Time 3; T4 = Time 4

Cost estimates differed by treatment stage for clinician-reported outpatient costs, *F*(10, 234) = 2.39, *p *= .01. At T3, early-stage patients had significantly higher outpatient costs at T3 (*M* = 2811.85) compared to later-stage patients (*M* = 2370.98), *F*(1) = 4.80, *p *= .03 (see Table 4 and Figure 4).

## 3. Discussion

Patients with DDs present to treatment with many complex psychiatric and safety-related issues. Many times, these patients have spent years in the mental health system receiving treatment based on inaccurate diagnoses such as schizophrenia. Once diagnosed correctly, DD patients can begin receiving appropriate, trauma- and dissociation-focused treatment, which can lead to reductions in symptoms and improvements in functioning (Brand et al., 2009; Brand, Classen, McNary, & Zaveri, 2009; Brand et al., 2013). However, years of treatment are typically required due to the chronicity and severity of these patients’ impairments (ISSTD, 2011). The current study sought to determine changes in costs of inpatient and outpatient mental health services utilization for DD patients receiving trauma- and dissociation-focused treatment. Patients’ and individual therapists’ reports of services were converted into USD to determine changes in cost over time.

We found decreases in longitudinal cost estimates based on patient-reported days of inpatient hospitalization in four out of six time comparisons, with small to mid-range effect sizes (*d *= .22–.33). The omnibus test of costs related to therapist-reported days of hospitalization was not significant. Most (two of three) of the analyses examining longitudinal cost estimates based on clinician-reported outpatient services indicated decreased outpatient costs over time. Specifically, significant decreases in utilization of outpatient sessions occurred between six and 18 months and six and 30 months in the study; these findings suggest a small effect of reducing outpatient costs over time (*d *= .14–.19).

We found a similar pattern of decreased costs with cross-sectional analyses of inpatient, and, to a lesser extent, outpatient costs. That is, in general, estimated costs tended to be higher among patients in the early stages of treatment compared to the later stages of treatment. Most of the patient- and therapist-reported inpatient costs were higher for early stage compared to later stage patients (i.e. three out of four comparisons based on patient reports, and two out of three comparisons based on therapist reports). At 18 months, cross-sectional cost estimates based on therapist-reported outpatient sessions showed significantly lower costs for patients in later stages of treatment compared to those in early stages of treatment. There were fewer cross-sectional differences in estimated outpatient treatment costs than there were in inpatient costs.

These findings demonstrate that DD patients estimated treatment costs gradually decrease during the course of treatment. This pattern of longitudinal and cross-sectional reductions in inpatient and outpatient costs was supported by both patients and therapists’ reports. DD patients who engage in trauma- and dissociation-focused treatment report a decrease in symptoms and their therapists report improvements in patient functioning (Brand et al., 2009, 2013). Such improvement is associated with less frequent self-injurious and suicidal behaviours (Webermann, Myrick, Taylor, Chasson, & Brand, 2015), which might have reduced the need for inpatient hospitalization and intensive outpatient treatment. Furthermore, ongoing treatment can assist patients in maintaining these improvements. In a six-year follow-up of the TOP DD study, only one patient out of 61 (1.6%) required inpatient hospitalization during the previous six months (Myrick et al., 2017). Based on the design of the current study, however, we cannot be certain about what caused these cost reductions. The lower treatment costs later in the study might be due to therapists following expert consensus treatment guidelines recommending that patients should be extensively stabilized before they begin intensive trauma-focused work. If adhered to, this implies that early in treatment clinicians might be more prone to hospitalizing patients who express suicidal thoughts than they are when the patient expresses such thoughts after 30 months of treatment. Alternatively, patients may be experiencing less suicidal ideation and impulses later in treatment.

DD experts recommend emphasizing safety and carefully pacing treatment so as not to overwhelm DD patients (Brand et al., 2012; Kluft, 1993; Myrick, Chasson, Lanius, Leventhal, & Brand, 2015). An early and consistent approach to safety might contribute substantially in gradually decreasing the costs and suffering of individuals with DDs; however, it was not the aim of the present study to examine the efficacy of the treatment. To examine efficacy and to determine if the treatment is responsible for reductions in cost and symptoms, future studies should include a waitlist control condition. Future (full economic) costs studies should examine cross-cultural variables that may impact treatment costs including referring practices, the availability of inpatient treatment, and length of stay. For example, Norwegian DD patients can attend a free three-month intensive inpatient trauma treatment programme (Jepsen, Langeland, Sexton, & Heir, 2014), whereas similarly long hospitalizations are rare in the U.S.

It is important that the findings be interpreted in the context of the study design. This study’s limitations included lack of a control group, small sample sizes that prevented analysing the inpatient and outpatient treatment costs for each individual treatment stage, the use of patient- and therapist-reported inpatient and outpatient services, limited contextual information about potential confounding variables, and high attrition. Other cost analysis studies have examined the economic impact of disability status, lost wages, suicidality, medications, and reduced productivity in the workplace; such costs were not gathered in this study but are important to assess in future research. The rate of attrition in this study was approximately 50%. Given the chronicity of the DD population and the length of the study (30 months), this attrition rate is not surprising or atypical for long-term treatment studies of mental illness (Mansfield et al., 2010). For example, only 20% of veterans remained in a national study of methadone maintenance at one year (Mansfield et al., 2010). Another study of veterans who had had repeated hospitalizations found that only 12% of the patients remained in outpatient treatment two years after being hospitalized (Bowersox, Saunders, & Berger, 2013). Furthermore, most trauma treatment studies exclude patients with complex and severe presentations including those with suicidality (Roberts, Roberts, Jones, & Bisson, 2016), although the present study did not exclude any patients based on symptomatology or chronicity (Brand et al., 2009). Attrition is higher in patients who have low incomes, receive government subsidies, and who struggle with substance abuse and/or serious psychiatric illnesses (Mancino et al., 2010), characteristics that were common in this sample (Brand et al., 2009). Although data regarding reasons for attrition were not collected, in cases where patients terminated their treatment with the therapist (and thus the study), therapists indicated reasons such as objective causes (e.g. relocation, transportation issues, financial difficulties), subjective causes (e.g. alliance issues), and treatment success (Myrick et al., 2017). Given the chronic course of DDs, our 30-month follow-up period is probably too short to show significant changes in long-term psychological symptoms, and thus a decline in costs.

We analysed the costs associated with treatment by therapists familiar with treating DD patients. The costs should also be explored for DD patients who are treated by therapists unfamiliar with DD treatment as well as the costs for DD patients who are misdiagnosed and treated for the wrong disorders (e.g. schizophrenia) to evaluate possible differences in cost estimates between these groups.

Overall, the current study found that DD patients who are engaged in specialized DD treatment showed decreased estimated costs for inpatient and outpatient mental health services over time. It is not clear whether it was the treatment that caused the reduction in the utilization of expensive inpatient and outpatient treatments over time for patients and the health care system. Investing in DD treatment research is warranted as a potential means of reducing patients’ suffering and treatment costs.

## Note

1. The authors converted all researchers’ findings to USD to facilitate comparisons with the present study’s findings.
